# Unravelling the Complexity of HNSCC Using Single-Cell Transcriptomics

**DOI:** 10.3390/cancers16193265

**Published:** 2024-09-25

**Authors:** Cristina Conde-Lopez, Divyasree Marripati, Moshe Elkabets, Jochen Hess, Ina Kurth

**Affiliations:** 1 Division Radiooncology/Radiobiology, German Cancer Research Center (DKFZ) Heidelberg, 69120 Heidelberg, Germany; jochen.hess@med.uni-heidelberg.de (J.H.); ina.kurth@dkfz-heidelberg.de (I.K.); 2 The Shraga Segal Department of Microbiology, Immunology and Genetics, Ben-Gurion University of the Negev, Beer-Sheva 84105, Israel; divyasree@bgu.ac.il (D.M.); moshee@bgu.ac.il (M.E.); 3 Faculty of Health Sciences, Ben-Gurion University of the Negev, Beer-Sheva 84105, Israel; 4 Department of Otorhinolaryngology, Head and Neck Surgery, Heidelberg University Hospital, 69120 Heidelberg, Germany

**Keywords:** HNSCC, single-cell RNA sequencing, tumor microenvironment

## Abstract

**Simple Summary:**

Head and neck squamous cell carcinoma (HNSCC) is a complex and varied cancer that is difficult to treat due to its biological diversity. Single-cell RNA sequencing (scRNAseq) offers a way to study HNSCC by examining individual cells within tumors. This technology helps identify different cell types and their roles in cancer development, diagnosis, and treatment. By understanding how various cells respond to treatments like chemotherapy and immunotherapy, scRNAseq can enhance the study of cancer biology and improve therapeutic outcomes. This review highlights the significant impact of scRNAseq on HNSCC research and its potential to advance the study of other cancers.

**Abstract:**

Background/Objectives: Head and neck squamous cell carcinoma (HNSCC) is a highly heterogeneous and the most common form of head and neck cancer, posing significant challenges for disease management. The objective of this review is to assess the utility of single-cell RNA sequencing (scRNAseq) in addressing these challenges by enabling a detailed characterization of the tumor microenvironment (TME) at the cellular level. Methods: This review compiles and analyzes current strategies that utilize scRNAseq and other single-cell technologies in HNSCC research. Results: For HNSCC etiology, scRNAseq allows for the construction of cellular atlases, characterization of different cell types, and investigation of genes and processes involved in cancer initiation, development, and progression within the TME. In terms of HNSCC diagnosis and prognosis, the resolution offered by scRNAseq enables the identification of cell type-specific signatures, enhancing prognostic models and disease stratifiers for patient outcome assessments. Regarding HNSCC treatment, scRNAseq provides insights into cellular responses to various treatments, including radiotherapy, chemotherapy, and immunotherapy, contributing to a better understanding of treatment efficacy and patient outcomes. Conclusions: This review highlights the contributions of scRNAseq to HNSCC research, addressing its cellular and biological complexity, and emphasizes its potential for advancing research and clinical practice in other cancer types.

## 1. Introduction

Head and neck squamous cell carcinoma (HNSCC) represents a significant burden in the global landscape of cancer, accounting for more than 90% of the head and neck cancers (HNCs). While advancements have been made in the treatment and prognosis of HNSCC in recent decades, the chances of a relapse within two years are above 50%, and the five-year overall survival (OS) rate remains below 50%, indicating substantial room for further improvement [1]. A major challenge in dealing with HNSCC lies in the high degree of intra-tumor and inter-tumor heterogeneity, which heightens the difficulty of administrating proper standard treatment [2,3].

In the last decade, transcriptomics has been based on bulk RNA sequencing, where all cells in a sample are sequenced together to produce one gene expression profile of the whole tumor. This is evidenced by collaborative initiatives like The Cancer Genome Atlas (TCGA) and The National Cancer Institute’s Clinical Proteomic Tumor Analysis Consortium (CPTAC), encompassing an extensive collection of tumor samples analyzed at various levels, including transcriptomics [4,5]. However, this approach fails to uncover the cellular sources of variation, as it combines multiple cell types in different developmental states, thereby obscuring critical molecular events and signals occurring within distinct cell subpopulations, which could be the key for therapeutical targeting [6,7]. Single-cell transcriptomics has emerged as a solution, providing a gene expression profile for each individual cell within the sample. This enables a detailed characterization of the tumor cells and their tumor microenvironment (TME) at the cellular level, shedding light on the intrinsic properties of distinct cell populations within one tumor, thereby facilitating the identification of therapeutic targets specific to different subpopulations [8].

The current literature illustrates the impact of single-cell transcriptomics through different HNSCC research aspects [2,9]. Moreover, scRNAseq has demonstrated its utility in other cancers, such as pancreatic ductal adenocarcinoma (PDA), where it helped to analyze cell population heterogeneity and phenotypic changes throughout disease progression, leading to the proposal of new targets for targeted treatment [10,11]. In breast and renal cell carcinomas, scRNAseq has identified gene expression differences between primary and metastatic tumors, highlighting prognostic biomarkers and potential therapeutic targets to prevent metastasis [12,13]. These examples underscore the relevance of single-cell transcriptomics across diverse cancer types and its impact on key research aspects: cancer etiology, diagnosis and prognosis, and treatment. These aspects will be discussed in more detail in the following sections relating them to the importance of scRNAseq analyses.

Cancer etiology involves fundamental research done to understand the variables and mechanisms underlying cancer initiation, development, and progression. HNSCC disease etiology is remarkably complex; it comprises a wide range of factors, ranging from the composition of the TME to genetic mutations and environmental exposures. In fact, one distinctive feature of HNSCC is the heterogeneity in the anatomical sites where it occurs, which further contributes to the overall complexity and variability of the disease compared to other cancer types. Cancer diagnosis and prognosis utilizes the insights obtained from fundamental research to identify the presence, location, and severity of the disease. This information is relevant in the search of key nodes that enable the stratification of patients according to their risk, the determination of proper treatment strategies, and the ability to monitor progress. Stratification is important because it allows for a more specific treatment plan according to the characteristics of the selected group. Lastly, modern cancer treatment uses a combination of basic knowledge about the cancer and its biomarkers or actionable targets to develop tailored treatment strategies and study the responses.

This comprehensive review evaluates the use of single-cell transcriptomics in HNSCC, emphasizing its contributions and the challenges it poses in advancing our understanding and management of this complex disease. These sections are intended to suggest possible future directions rather than assert definitive clinical conclusions.

## 2. HNSCC Etiology

Single-cell transcriptomic data can be used to unravel and provide a deeper understanding of the biological mechanisms and factors driving cancer initiation, development, and progression. Fundamental research is essential to lay the foundation on which optimized cancer diagnosis, prognosis, and treatment will rest. The use of single-cell analysis introduces a new layer of complexity to our understanding of the TME, taking the information to the cellular level and allowing examination of the changing patterns in tumor cells and their surrounding microenvironment [14].

**Figure 1:**
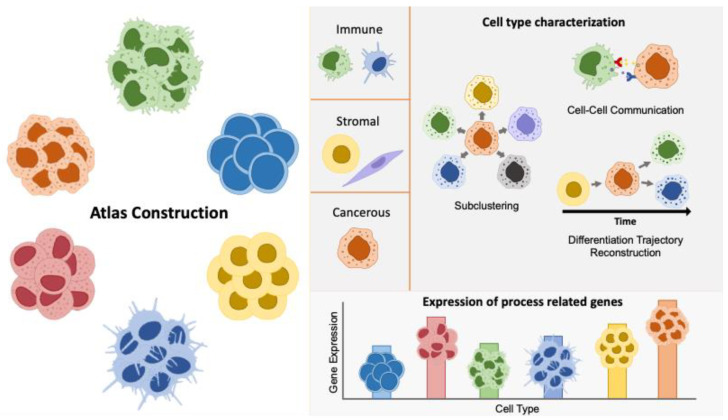
Illustration of strategies utilized in single-cell transcriptomics to address cancer heterogeneity. The figure highlights methods such as atlas construction, sub-clustering, cell type characterization, cell–cell communication analysis, differentiation trajectory reconstruction, and expression profiling of process-related genes. The expression of process-related genes enables the evaluation of genes involved in critical cellular functions and pathways across different cell types, as distinguished by single-cell techniques, providing insights into how these gene activities drive cancer progression and identifying the specific cell types through which these processes occur. These approaches collectively enhance our understanding of the diverse cellular components and interactions within the tumor microenvironment.

HNSCC represents a biologically diverse disease that exhibits a substantial level of histological and molecular heterogeneity. This includes intra-tumoral heterogeneity, which refers to the presence of diverse subpopulations within a single tumor sample, and inter-tumoral heterogeneity, which describes the differences observed between tumor samples from distinct anatomic sites within the same patient, as well as between different patients or tumor types [9,15]. In the current literature, several strategies have been proposed to give further insight into cancer heterogeneity using single-cell transcriptomics for a variety of cancer types. These approaches include atlas construction, cell type characterization, and expression of process-related genes (Figure 1).

### 2.1. Atlas Construction

One of the approaches to explore the complexity of HNSCC is the generation of HNSCC cell atlases, resulting in cell-type maps or collections of cell-specific gene expression profiles. The application of these atlases allows for an in-depth exploration of HNSCC cellular diversity across sites, states, and patients, trying to reach the highest degree of resolution that is financially and technically possible.

Puram et al. published the first HNSCC atlas, in which ~6000 cells from 18 HNSCC patient, including primary tumors and lymph node metastasis samples, were analyzed to allow for comparisons of tumor progression [16]. This study aimed to investigate intra-tumoral heterogeneity, defining different cell types composing the TME across patients and differentiating between malignant and non-malignant cell types. The study also encompassed inter-tumoral heterogeneity, with results showing similarities in the expression profiles of defined cell types throughout different patients. In a more recent study, the focus shifted to profiling oropharyngeal squamous cell carcinoma (OPSCC), including both HPV-positive and HPV-negative tumors, expanding the cell count to 70,970. This comprehensive analysis revealed extensive cellular diversity within and between tumors and uncovered a subset of malignant cells in HPV-positive tumors lacking detectable HPV expression, which also exhibited reduced HPV-associated phenotypes [17]. Another example is the HNSCC atlas reported by Kürten et al. that provides a broad overview of immune and non-immune cells in HNSCC [18]. The samples include matched CD45+ peripheral blood leukocytes (PBL) as control samples and tumor-infiltrating leukocyte (TIL) samples, as well as CD45− non-immune cells obtained from primary tumors of 15 HNSCC patients. In this study, an in-depth characterization of tumor populations and differences in inflammation levels in HPV+ versus HPV− HNSCC was performed. Moreover, HPV viral gene expression was shown to be traceable in single cells. The breadth of the dataset allowed the characterization of multiple cell types, with a particular focus on epithelial cells and CD8+ T cells and their dysfunctional state, which has been targeted as one of the main effectors of anti-cancer immunity. In another HNSCC atlas presented by Choi et al., the aim was to profile the progression of HNSCC cell types and cell–cell interactions with samples ranging from normal tissue to metastatic tumors in the lymph node, including precancerous leukoplasia and primary tumors [14]. Immune cells, specially Tregs and CD154-CD4+ T cells infiltration, were positively correlated with tumor progression. The proportional changes in cell types were proven to reflect cancer progression according to microenvironment alterations. HNSCC atlases can have a fewer number of samples but a much higher cell number per sample. In the atlas proposed by Song et al., two samples from the larynx were analyzed, and ~14,000 cells were retrieved, allowing for the characterization of the immune compartment and the assessment of tumor cell heterogeneity among the samples [19]. The largest patient cohort for scRNAseq in HNSCC is presented in the atlas by Bill et al., profiling 52 fresh tumor tissues from 51 patients, encompassing 187,399 cells and a wide range of clinical characteristics. By addressing the heterogeneity of HNSCC, the study revealed significant insights into tumor-associated macrophage (TAM) polarization and its impact on the TME, highlighting the potential for new therapeutic interventions across multiple cancer types [20].

In summary, the broadness of the data obtained from SC atlases enables an unprecedent amount of information that can be extracted from a single experiment. Moreover, the importance of proper clinical annotation of the patients is demonstrated in the wider range of questions that well-annotated data can address. Such annotations can include factors like sex, HPV status, or exposure to environmental factors like alcohol or tobacco. The imperative for research transparency has propelled these studies to render their data openly accessible, thereby facilitating utilization by fellow researchers who might be constrained in their capacity to generate SC data independently or whose inquiries could be addressed using preexisting datasets. The development of integration methods that assess biological variation across samples has created opportunities to unify data from different studies. This allows for the creation of a ‘universal atlas’ as a comprehensive reference map for HNSCC, overcoming current limitations such as the small number of cases processed per study. A list of available HNSCC scRNAseq datasets is provided in Table 1.

The atlases described primarily focus on identifying differences in the proportion and behavior of cell populations within tumors. These studies offer insights into how specific cell types, like tumor-associated macrophages (TAMs) and CD8+ T cells, contribute to disease progression. This detailed characterization helps in understanding the cellular causes of HNSCC and could lead to more targeted treatments. The implications of these findings will be further discussed in the following sections.

### 2.2. Cell Type-Specific Characterization

A different approach to unraveling the HNSCC characteristics is to focus on specific cell subsets to gain a deeper understanding of their dynamics, location, and interactions. Such an approach has proven highly relevant for characterizing the TME and identifying targets for treatment. Several studies have pursued this task as one of the analyses performed after generating an atlas [19,21,29]. Other publications use publicly available atlas data to explore unstudied subpopulations, such as subtypes of cancer-associated fibroblasts (CAFs) or tumor cells [30]. These publications may supplement the study with data of their own [31]. A third option is the generation of new data that are specifically oriented toward a particular subpopulation, for instance B cells or CD8 T cells selected through cell sorting [24,25,32].

In the current literature, the main methods for analyzing scRNAseq data include sub-clustering of a designated cell type to distinguish cell states based on expression profiles. This is followed by techniques like differential expression (DE) or pathway analysis, cell–cell communication (CCC) analysis to identify potential intercellular interactions and communication pathways, and differentiation trajectory reconstruction (DTR) to trace the developmental course of related cell states. Two main cell compartments have been explored using this methodology in the literature, the tumor cell compartment and the immune compartment.

#### 2.2.1. Tumor Cell Characterization and Crosstalk with Stromal Compartments

Tumor cells, which possess a proliferative advantage over neighboring cells, along with their surrounding stromal cells are a key player in cancer development. The significant heterogeneity observed among tumor cells, as well as among the components of the stromal compartment, may lead to differences in morphological and physiological features of HNSCC [19]. A comprehensive characterization of these subpopulations is needed to understand their behavior and devise effective therapeutic strategies to specifically target them. One of the main limitations of working with tumor cells using scRNAseq technologies is proper cancer cell annotation. Although some methods use manual selection of clusters based on the expression of marker genes or their copy number variation status (CNV), novel machine learning-based methods are emerging that automatize the process of tumor cell selection [33,34].

In order to gain a deeper understanding of a cell type, an initial step is the employment of sub-clustering methods to find subtypes or sub-states that may arise from different cancer stages and interactions with the environment. One study focused on sub-clustering tumor cells, and five sub-clusters were identified based on cluster marker genes: keratinocyte-like, proliferative, immortal, metastasis, and immune cell clusters [19]. Clusters were labelled according to the Gene Ontology (GO) pathways that were enriched in each sub-cluster. In another study, tumor cells were detected as a sub-cluster of epithelial cells and further divided into three cluster based on cluster marker genes with previously characterized functions: partial epithelial–mesenchymal transition, cycling G2/M, and cycling S [30]. A cancer stem cell (CSC) sub-cluster was selected among 12 cancer cell sub-clusters due to an enrichment in stem cell marker expression, such as CD44, CD98, CD47, and CD276. While CD44 is an indispensable marker for CSC selection in other cancers like breast or colorectal cancer, this study found that OCT4, SOX2, and NANOG were more suitable for identifying CSCs in HNSCC [31].

DTR analysis can be useful to understand the chronological evolution of a cell population. In the five sub-cluster study by Song et al., the trajectory analysis showed the proliferative sub-cluster at the beginning of the trajectory, branching into immortal tumor cells or keratinocytes-like tumor cells, creating a timeline for the development of cancer cell types. Keratinocytes-like and proliferative tumor cells were observed to have distinct spatial location, with the latter being associated with worse patient prognosis [19].

CCC can be employed to understand how tumor cells interact with their surroundings. For instance, such analyses have shown that TNFSF12 (TWEAK, an apoptosis-inducing ligand) in CAFs has an opposite expression pattern in primary tumors, depending on whether they later metastasize or not. High expression of TNFSF12 is linked to cancer progression through proliferation, invasion, and EMT in nearby tumor cells and is associated with lower survival probability in HNSCC [30]. Sub-clustering of CAFs in HNSCC has shown differences in gene expression profiles, with the presence of specific sub-clusters being associated with metastasis and bad prognosis. These findings highlight the relevance of CAF subtypes in HNSCC [30,35]. CAF subtypes are extensively discussed by Lavie et al. [36]. CSCs and myeloid cells were found to interact through cell–cell communication analysis; this suggested that CSCs contribute to immune escape signaling by promoting maturation and differentiation of myeloid cells [31]. HPV status assessment was performed at the single-cell level in tumor cells, and CCC analysis correlated HPV+ tumor cells with infiltrated T cells, which may account for the difference in prognosis according to HPV status [37].

#### 2.2.2. Immune Landscape

The immune component of the TME is responsible for tumor-related humoral and cellular immune response, including immune surveillance and tumor growth control [38]. HNSCC cells have proven to be highly evasive to the immune system as well as having immune suppressive features [39]. However, the HNSCC immune landscape has a high degree of plasticity. HPV+ tumors exhibit a more immune-active or “hot” phenotype, while HPV− tumors are usually characterized by a more immune-suppressed or “cold” phenotype [40]. Patients with a colder tumor phenotype exhibit worse prognosis and less response to immunotherapy [41]. Therefore, classifying a tumor as having a hot or cold phenotype can be important for informing treatment strategies. Dissecting the immune component at the single-cell level may help in tailoring the treatment approach for each patient.

A sub-clustering of T cell populations by Chen et al. identified fourteen T cell sub-clusters that were further characterized according to their cluster marker gene expression. The comparison between states, including normal tissue versus tumor tissue, allowed for the identification of a higher proportion of exhausted T cell sub-states with less cytotoxic potential in tumor samples, making the blockage of their immunosuppressive role a target for immunotherapy [32]. A more specific study on HPV+ CD8+ T cells allowed for their classification in three sub-clusters, each one enriched in cluster markers for a different developmental stage (stem-like, transitory, and differentiated stages) [25]. In a different study by Huang et al. four sub-clusters of macrophages were detected. DE analysis between these sub-clusters also allowed for their classification according to developmental stage in early, transforming, and mature clusters [42]. Sub-clustering of B cells from healthy and HNSCC donors allowed the differentiation of six B cell sub-clusters. Assessment of the expression of multiple transmembrane cytotoxic tumor necrosis factor (TNF) super family (TNFSF) ligands based on the different sub-clusters showed differential expression in all these sub-clusters. These findings suggest that specific sub-clusters of tissue-resident B cells, especially germinal center B cells, might possess elevated intrinsic cytotoxic capabilities compared to their counterparts in the circulatory system [29].

Comparisons between carcinogen-mediated and virus-driven immune landscapes in HNSCC revealed notable differences in transcriptional profiles, relative composition, and developmental trajectories, primarily within CD4+ conventional T (CD4+ Tconv) cells, B cells, and myeloid cells. These differences are hypothesized to arise from the activation of the innate immune system mediated by the presence of the virus [21]. B cell-related pathways seemed to be important in HPV+ HNSCC in contrast to HPV− tumors, as well as genes revealed by DE and survival analysis. Upregulation of AREG and TGFBI (markers of plasmacytoid dendritic cells (pDCs)) was associated with worse survival, whereas downregulation of CD27, CXCR3 (expressed in T cell-related cells), MS4A1, and CD19 (expressed in naive B cells) was related to improved survival [43]. In another recent study, sub-clustering of macrophages in eight sub-clusters allowed for a more intricate classification of this cell type than the classical M1-like (anti-tumor) or M2-like (pro-tumor) macrophage classification described in the literature. This study validated that the infiltration ratio of M1-like sub-clusters was increased in HPV+ tumors, and a TCR+ tumor-promoting subpopulation described in the previous literature was also detected, showing how SC resolution allowed for a deeper characterization of these sub-populations [44].

DTR analysis in CD8+ T cells revealed a similar behavior between HPV+ and HPV− tumors, suggesting a potential similarity in the response of CD8+ T cell therapy across these distinct tumor types [21]. The same analysis in a broader T cell population from healthy and primary tumor samples demonstrated different developmental branches according to the “T cell activation signal” the initially non-functional T cells were receiving [32]. Macrophage DTR analysis from HPV+ versus HPV− tumors demonstrated similarities in the developmental trajectories between the two groups [44]. DTR analysis validated developmental trajectories from DE-derived stage-related genes. In a study on HNSCC-associated TAM (tumor-associated macrophage) populations, three sub-clusters were categorized by developmental stages using cluster marker genes. Remarkably, the DTR analysis confirmed the identical trajectory, reinforcing its validity [25,42].

Cell–cell communication analysis showed an increased cross-talk in the immune component of the TME versus non-tumor control samples [21]. CCC analysis applied to TCR+ versus TCR− macrophages from tumor samples showed differences in immune cell interactions, which suggest a reason for their differences in aggressiveness [44].

In summary, in-depth investigations into distinct cell types offer a wealth of insights into their behavior under specific conditions, developmental trajectories, and intercellular interactions. This could serve as a resource for identifying biomarkers and targets directed toward distinct cell types implicated in specific conditions, surpassing bulk methods in resolution. It is important to recognize that tumor cell populations and their microenvironment are constantly changing. HNSCCs are characterized by intra-tumoral heterogeneity with different subpopulations of cancer cells possessing distinct genetic, phenotypic, and behavioral traits. These differences contribute to the complexity of tumor behaviors, including metastasis or treatment resistance. Tumor heterogeneity arises from genetic variation within the tumor, such as CNVs, somatic mutations, and chromosomal instability, leading to distinct subclones of tumor cells. Clonal evolution, in which tumor cells acquire additional mutations over time, also generates diverse subclonal populations, leading to the dominance of certain clones with more aggressive characteristics [45]. Epigenetic plasticity, including DNA methylation changes and histone modifications, allows cancer cells to reversibly switch between different phenotypic states. Microenvironmental influences, such as hypoxia, nutrient availability, and cellular interactions with stromal and immune cells and other cancer cell subclones, exert selective pressures that shape tumor heterogeneity. The interplay between these tumor cells and the surrounding cell populations actively shapes the composition of the tumor microenvironment. However, scRNAseq captures only a snapshot of this dynamic process and lacks the spatial context necessary to fully understand these interactions. The functional characteristics of tumor cells are often aligned with the hallmarks of cancer, such as accelerated proliferation, resistance to cell death, immune evasion, invasion, and migration. scRNAseq is able to capture these often small subpopulations of tumor cells that carry genetic hallmarks of cancer, such as cells with distinct gene expression patterns that facilitate migration and lead to metastasis. The emphasis on studying specific subpopulations rather than the bulk of the tumor may pave the way for better biological understanding. Longitudinal studies monitoring tumor and stromal subpopulations and their gene expression over time during and after tumor treatment may be informative to targeting specific resistance subpopulations of tumor cells. Understanding the spatial and temporal dynamics is critical to understanding the interplay between genetic profiles and functional characteristics of different tumor subpopulations that significantly impact cancer progression and treatment outcomes. scRNAseq essentially helps to unravel the genetic profiles at a specific tumor state. To achieve a comprehensive understanding, validation using wet lab experiments and correlation with complementary data sources, such as functional and spatial annotations, patient survival data, bulk RNAseq, histology, or spatial transcriptomics, is imperative.

### 2.3. Expression of Process-Related Genes

scRNAseq provides insight into the study of genes relevant to the molecular and cellular mechanisms underlying HNSCC. Such data have been key in characterizing processes like ferroptosis and cuproptosis, where CSCs were identified as the core cluster related to ferroptosis, and epithelial cells showed high expression of CDKN2A, a gene associated with copper-induced cell death [46,47]. Utilizing scRNAseq data from atlases, which mitigates the high economic burden of generating new data, allows researchers to answer pivotal questions. Online interactive tools like CancerSEA and TISH2 facilitate these analyses by linking gene expression to functional states across various cancer types [48,49]. For example, CancerSEA was used to investigate perineural invasion (PNI) in HNSCC, revealing that fibroblasts, which exhibit high expression of PNI-related genes, play a significant role in processes like EMT, metastasis, and invasion [50]. Furthermore, the cornichon family AMPA receptor auxiliary protein 4 (CNIH4) has been identified as a key cancer marker associated with stemness, cell cycle, DNA repair, invasion, and proliferation, with high expression in tumor cells [51]. Additionally, scRNAseq data have shown that genes involved in mitochondrial dynamics and store-operated calcium channels are heterogeneously expressed across cell types, highlighting fibroblasts and cancer cells as potential cancer drivers [52]. Genes like APOBEC3B, APOBEC3C, and YTHDC1, highly expressed in tumor cells, were linked to tumor stemness and correlated with markers such as SOX2 and BM1 [53,54]. SEC11A, characterized as an oncogene and valuable prognostic biomarker, showed expression across multiple cell types, particularly correlating with immunosuppressive immature myeloid cells (MDSCs) [55]. Chloride intracellular channel 4 (CLIC4), a tumor suppressor, was downregulated in tumor cells when compared to tumor-associated fibroblasts and endothelial cells, supporting its role in growth arrest, differentiation, and apoptosis [56]. Moreover, scRNAseq has been used to characterize the expression of genes involved in tumor growth, angiogenesis, invasion, and metastasis through pathways like the CXCL12/CXCR4 axis [57]. By integrating these insights, scRNAseq data provide a comprehensive understanding of the cellular and molecular landscape of HNSCC, potentially uncovering new therapeutic targets and biomarkers.

## 3. HNSCC Diagnosis and Prognosis

**Figure 2:**
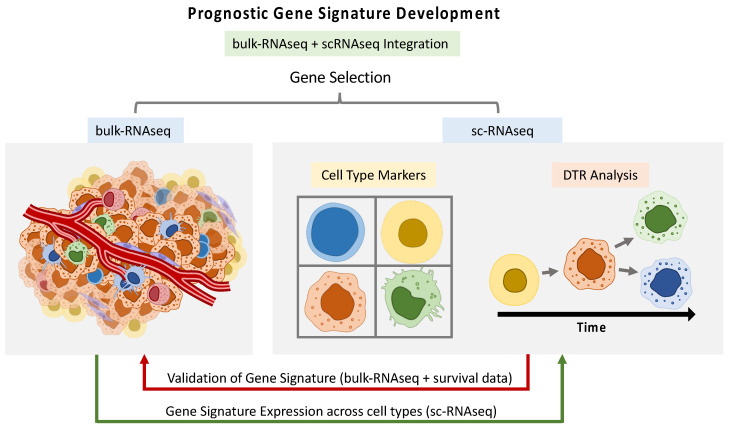
Workflow illustrating the integration of single-cell RNA sequencing (scRNAseq) and bulk RNA sequencing (bulk RNAseq) data for the development of prognostic gene signatures. This process can include gene selection, differential transcriptome (DTR) analysis, and validation using survival data. The integration allows for comprehensive multi-level data analysis, enhancing the utility and accuracy of prognostic models by leveraging detailed cellular insights from scRNAseq alongside broader bulk RNAseq data.

Despite advances in current treatments for HNSCC, patients with advance stages of the disease only have a 50% five-year survival rate, and, in the past few decades, there has been little improvement in this regard [58,59]. Novel therapies targeting immune checkpoints, such as inhibitors of the programmed cell death-1 (PD-1)/PD-1 ligand (PD-L1), have proven to be effective in only a small sub-group of patients. In order to guide more personalized treatments, strategies that help in the prediction of HNSCC progression and the selection of molecular biomarkers related to patient prognosis are highly important [60,61]. The deep resolution offered by SC technologies allows for a more refined selection of cell type-related signatures, which could provide insights for the construction of prognostic models and disease stratifiers. The information can be transferred from SC data to bulk data or vice versa in diverse ways, paving the way for multi-level data integration and allowing for maximal usage of all available data (Figure 2).

Prognostic gene signatures established from bulk RNAseq analysis can be further characterized using scRNAseq analysis. Eight methylation/autophagy-related genes (MARGs) and two immune infiltrating cell types were selected to build a prognostic risk scoring (pRS) system. Clustering analysis of the eight-gene pRS divided single cells into high- or low-risk cells, with fibroblasts being mainly present in the high-risk cluster. Given heterogeneity in the expression of the eight genes used to construct the pRS system across cell types, a neural network-based deep learning model was built based on scRNAseq data that was able to infer cell types [60]. Due to the prognostic value of FGFR signaling, hypoxia, and glycolysis, a six-gene fibrosis–hypoxia–glycolysis-related prognostic classifier was constructed based on bulk RNAseq. Through SC analysis, the expression of this gene signature was found to be higher in monocytes; this cell type also showed a higher infiltration in HNSCC patient samples than in healthy donors, which points to its association with poor prognosis [61]. Also, SC data were used in order to verify the relation between genes and the six-gene matrisome-associated prognostic model. A risk score for each cell was calculated, and higher risk was associated with tumor cells compared to the non-tumor cells [62].

A different approach to integrating SC and bulk RNA seq data relays the importance of particular cell types to cancer development and outcome. In a study about mast cells (MCs), a cell type that plays a pro-tumorigenic role by promoting several cancer-inducing functions, MC cluster marker genes extracted from SC analysis were selected to construct a MC-related prognostic model using bulk RNAseq data [3]. HPV+ CD8+ T cell cluster marker genes were selected using DE analysis comparing HPV+ and HPV− CD8+ T cell populations established using SC analysis. These genes were subsequently utilized to establish a prognostic gene signature able to divide HPV+ patients in high- and low-risk groups [58]. In a further study, a CAF-related gene signature was developed based on cluster marker genes associated with poor prognosis from a CAF subpopulation identified using SC analysis. The high-risk group, selected in terms of OS, was associated with low levels of anti-tumor immune cells and less sensitivity to conventional chemotherapy and immunotherapy, highlighting immune cell infiltration as a main driver of the risk differences [35]. In a different study, to assess the prognostic value of CD4+ T follicular helper (TFH)-related genes, the TFH-related signature was analyzed using bulk RNAseq data paired with survival analysis, which found that its enrichment was associated with better progression-free survival (PFS) [21]. Deconvolution algorithms can be utilized to computationally estimate cell-type proportions in bulk expression data using information extracted from SC data [63]. For example, TCGA bulk RNA seq data were deconvoluted using SC data, giving a cell fraction score for each of the ten cell types detected in the SC dataset. Using this information, an eight-gene prognostic model was constructed using genes correlating with a higher CD8+ T cell content. Risk-divided populations were then characterized. The low-risk group was identified as a more immune-active or hot type than the high-risk group, with the low-risk group representing the better responders to immunotherapies [58].

The prognostic value of cell differentiation-related genes (DRGs) from scRNAseq trajectory analysis has proven useful for HNSCC molecular stratification and therapy selection [64]. For example, a study identified 159 prognostic DRGs, resulting in three subtypes—active stroma, active metabolic, and active immune—each with distinct therapeutic implications [7]. DTR in SC data also revealed LN-related genes, leading to a five-gene prognostic model that identified molecular subtypes and informed therapeutic decisions [65]. Finally, CCC analysis in scRNA-seq identified key interactions across HNSCC samples, leading to the generation of a five-gene model by multifactorial stepwise regression, highlighting the importance of these interactions in cancer progression [66].

## 4. HNSCC Treatment

The final step in HNSCC cancer research is to actively attack the cancer using a selected treatment. Treatments for HNSCC with a curative intent includes tumor resection, radiotherapy, chemotherapy, or their combination [67]. However, tumor relapse has still an incidence of about 50% within 2 years, and the 5-year survival rate remains quite unfavorable with rates of less than 50% [1]. In the last decades, due to the appearance of immunotherapy and better selection of target groups, treatment of HNSCC has steadily improved [68]. Inter-tumoral heterogeneity in HNSCC is among the reasons for the unfavorable outcomes, accounting for the variability in therapeutical responses among patients [69]. The use of scRNAseq contributes to the understanding of specific cellular responses to treatments, including radiotherapy, chemotherapy, and immunotherapy, which could, in turn, lead to improvements in treatment approaches and patient stratification.

Single-cell analysis has the potential to greatly improve the monitoring of treatment responses in longitudinal human studies. It allows tracking of various cell populations at the clonal level, opening a new way to investigate treatment effects. Although this approach is still in its early stages, a few studies have started this exploration, with more being expected in the future. In the areas covered by this review, the use of SC analysis for monitoring patient treatment response remains relatively untouched. In the two human studies described in this section, the proposed strategy involved comparing primary tumor samples before and after treatment to observe changes in cell populations in response to treatment. Using SC analysis on samples taken before and after treatment with nivolumab, CAFs were identified as the cell population exhibiting the greatest changes in abundance. This study led to the identification of a CAF subpopulation related to immunoactivation, the so-called T cell-stimulating CAFs (tsCAFs). This knowledge allows for the development of strategies to force CAF differentiation toward the proinflammatory tsCAF phenotype rather than the immunosuppressive phenotype, leading to an improvement in tumor response to immunotherapy [22]. Additionally, SC analysis of samples treated with induction chemotherapy (ICT), chemotherapy administered before surgical removal of the tumor, further highlighted the importance of CAFs in tumor treatment. In the study, CAFs showed upregulation of EMT-related pathways induced by chemotherapy, recognizing them as drivers of response and potential targets during therapy. A limitation presented in this study is that samples derived from only one patient were used [70].

Other examples of strategies followed to study HNSCC response to treatment using scRNAseq can be found in preclinical studies. In some of these studies, analysis is conducted by comparing before and after treatment states. In contrast, in other studies, silencing or knock-out models of target proteins are performed as way to mimic the treatment effect. In all cases, a pre-treatment reference is utilized to track changes in a cell population’s behavior. B cells from HNSCC tumor samples induced by subcutaneous injection of B16-OVA and AT-84-E7-OVA cells, which reflects HPV+ HNSCC in mice, were analyzed before and after anti-PD-L1 immune checkpoint blockade (ICB), radiotherapy, and ICB combined with radiotherapy. A higher percentage of germinal center (GC) B cells in ICB combined with radiotherapy was detected. GCs are the hallmark of B cell-mediated adaptive immunity, so their presence is a sign of treatment effectiveness [71]. Evaluation of IFNAR-1 blockade as a potential therapy was assessed using SC analysis of samples from HNSCC tumors generated by subcutaneous implantation of empty vector or shIfnar-1 MOC2-E6/E7 cells in mice. The study of cell population composition led to the conclusion that IFNAR-1 blockade in tumor cells expands stem-like effector T-cells, restricts MDSCs, and reduces tumor burden, demonstrating its validity as a therapeutic option [72]. The effect of the ICB therapies anti-PD1 and anti-CTLA4 on TILs of different murine oral carcinoma (MOC) models was evaluated using scRNAseq analysis. The study focused on the T cell compartment, specifically the CD8+ T cell sub-cluster. In samples showing the best response to the treatment, T cells had differentiated from naive/memory- like states to PD1+ activated states, unraveling the tumor characteristics necessary for successful anti-PD1/anti-CTLA4 therapy [73]. To study the therapeutic effects of tRNA m7G methyltransferase METTL1 blockade, scRNAseq analysis of samples from Mettl1-knockout (KO) mice with 4NQO-induced HNSCC was performed. Alterations in the immune landscape were shown to occur in Mettl1-KO mice, with a decrease in CD4+ T exhaustion and Tregs, making METTL1 blockade a suitable treatment strategy [74].

Recent studies in other cancer types, such as endometrial and pancreatic cancers, have demonstrated the significant potential of combining scRNAseq with spatial transcriptomics to unravel the complexities of the TME and potentially enhance therapeutic strategies. For instance, in endometrial cancer, this integrated approach has revealed distinct immune cell populations influencing the response to anti-PD-1 therapy, while in pancreatic cancer, it has provided deeper insights into the spatial dynamics of CAFs and their role in therapy resistance [75,76]. Applying similar methodologies to HNSCC may provide useful insights into the spatial organization and interaction of cell types within the TME, which could contribute to a better understanding of therapeutic resistance and response.

## 5. Outlook

The advancement of scRNAseq technology still faces significant challenges such as comparability of results. One of the main obstacles is the accurate characterization of cell types and states, given that cells exist in a dynamic state of continuous change, while scRNAseq captures only a snapshot of this process. Cell labeling, which involves selecting the correct cell type label under which each cell will be allocated, is a critical step in scRNAseq analysis and directly impacts the obtained results. However, various studies employ their own definitions of cell types and states, making it difficult to integrate and consolidate the findings [15]. Even more, labelling can be limited by the current biological knowledge, finding cells in states that have not yet been defined [44]. The development of automated tools, such as AI-driven labelling tools and correct pruning and curation of the data, offers a promising way to achieve standardization of results derived from scRNAseq analyses [77]. For instance, the scGate labelling tool effectively filters and annotates single-cell RNA sequencing data, enhancing the accuracy and reproducibility of cell type identification [78]. Nevertheless, the proliferation of tools can also pose some challenges. With many tools emerging daily, each claiming to offer improved results, researchers face difficulty in selecting an appropriate analysis method. As a result, each study tends to have its own unique analysis approach. Community efforts like “Single-Cell Best Practices” and “scRNA-tools” aim to combine and consolidate knowledge and provide general guidelines that could also contribute to standardization in the analysis process and improve result consistency [79,80]. Additional issues associated with scRNAseq include the limited depth of data, confined to the transcriptome dimension. Although in some cases the transcriptomic information is paired with cell surface markers, due to cells being sorted by fluorescence-activated cell sorting (FACS), the overall information remains quite limited. To overcome these limitations, the integration of complementary SC technologies, such as spatial technologies that trace transcripts to their localization within tissue or SC genomics or epigenetics, can provide a multi-layered insight alongside scRNAseq data [81].

In addition to these challenges, it is essential to acknowledge that the TME is composed of various cell types, some of which, like adipocytes and neural cells, are less abundant and have not been extensively investigated in the literature, particularly using SC methods. These less studied components of the TME could be significant sources of novel therapeutic targets for prognosis and treatment. Further research is necessary to deepen our understanding of their roles within the TME, as they may play critical roles in tumor progression and patient outcomes.

Costs and manpower limitations are still relevant in the SC field. SC techniques are complex, requiring qualified professionals, thereby adding to the already substantial expenses associated with the technology, including material costs [6]. A significant advantage of these technologies is that available public data can be used to address a wide range of research questions, potentially reducing project costs. Furthermore, computational methods can facilitate the reusability of the previously existing data. For example, the practice of pseudobulking, which involves converting scRNAseq data into bulk RNAseq data, allows the use of existing tools established for bulk analysis in scRNAseq. Pseudobulking enables the extraction of multiple smaller and more specific datasets from a larger dataset and it offers the possibility of increasing the versatility of the data by generating subsets that are tailored to address distinct research questions or objectives [82]. SC data integration tools are also valuable for effectively utilizing existing data by combining information from different sources, patients, and studies. The reason for this is that these integration tools can assess technical biases between samples while preserving the relevant biological bias necessary for accurate analysis [83,84].

One of the emerging hot topics in cancer research is cancer prevention, which remains relatively unexplored in HNSCC. scRNAseq has the potential to significantly contribute to this field by providing a detailed understanding of the earliest cellular changes that occur during cancer initiation. For instance, scRNAseq could be used to identify and characterize premalignant lesions at the single-cell level, revealing key molecular pathways involved in the transition from normal to malignant states. Additionally, scRNAseq can be used to further study and validate preventive strategies, such as HPV vaccination [85], by analyzing the cellular and molecular impact of these strategies on the organism. By elucidating the mechanisms through which prevention strategies impede cancer development, scRNAseq can help refine and optimize prevention protocols, ultimately contributing to more effective cancer prevention strategies.

## 6. Conclusions

The use of scRNAseq technologies provides a detailed view of cellular diversity, which can be crucial in deciphering cell-dependent processes. Due to the high heterogeneity of HNSCC, the application of SC technologies is quite promising, as was shown in this review. Further questions to be addressed using these types of methods include processes that can be traced back to each individual cell. An example of this is the effect of the HPV virus on cellular behavior and the relevance of cell type and state in this context. Similarly, the study of sex chromosome behavior, including their presence, expression, and function across different cell types and states, can uncover critical factors that contribute to the composition of and interaction with the TME, ultimately driving cancer initiation, establishment, and progression [86,87]. Therefore, to fully exploit the potential of scRNAseq technology, it is essential to assess the specific requirements of each research question and use complementary tools that provide a more throughout understanding of cellular processes. By facilitating comprehensive characterization of distinct cellular behaviors, scRNAseq enables a deeper comprehension of HNSCC, thereby allowing enhanced patient stratification and eventually personalized treatment selection.

## Figures and Tables

**Table 1:** Summary of HNSCC scRNAseq datasets with human samples available in GEO. This table lists the datasets by accession number, publication year, authors, data type, tissue types analyzed, the number of samples, and the number of cells.

Accession Number	Year	Publication	Data Type	Tissue Types	Sample Number	Cell Number
GSE234933	2023	Bill, R.; Wirapati, P.; Messemaker, M.; Roh, W.; et al. CXCL9:SPP1 macrophage polarity identifies a network of cellular programs that control human cancers. *Science* **2023**, *381*, 515–524 [20]	scRNAseq	Primary tumor Local recurrence Distant metastasis	52	87,399
GSE182227	2022	Puram, S.V.; Mints, M.; Pal, A.; Qi, Z.; et al. Cellular states are coupled to genomic and viral heterogeneity in HPV−related oropharyngeal carcinoma. *Nat. Genet.* **2023**, *55*, 640–650 [17]	scRNAseq	Primary tumor Normal tissue	24	70,970
GSE139324	2019	Cillo, A.R.; Kürten, C.H.L.; Tabib, T.; Qi, Z.; et al. Immune Landscape of Viral- and Carcinogen-Driven Head and Neck Cancer. *Immunity* **2020**, *52*, 183–199.e9 [21]	scRNAseq	Peripheral and intra-tumoral CD45+ populations	63	131,224
GSE164690	2021	Kürten, C.H.L.; Kulkarni, A.; Cillo, A.R.; Santos, P.M.; et al. Investigating immune and non-immune cell interactions in head and neck tumors by single-cell RNA sequencing. *Nat. Commun.* **2021**, *12*, 7338 [18]	scRNAseq	Primary tumor Peripheral blood leucocytes	51	134,606
GSE103322	2017	Puram, S.V.; Tirosh, I.; Parikh, A.S.; Patel, A.P.; et al. Single-Cell Transcriptomic Analysis of Primary and Metastatic Tumor Ecosystems in Head and Neck Cancer. *Cell* **2017**, *171*, 1611–1624.e24 [16]	scRNAseq	Primary tumor	18	5902
GSE181919	2022	Choi, J.H.; Lee, B.S.; Jang, J.Y.; Lee, Y.S;. et al. Single-cell transcriptome profiling of the stepwise progression of head and neck cancer. *Nat. Commun.* **2023**, *14*, 1055 [14]	scRNAseq	Primary tumor Normal tissue Leukoplakia Lymph node metastasis	37	54,239
GSE173647	2022	—	scRNAseq	Primary tumor	2	13,903
GSE195832	2022	Obradovic, A.; Graves, D.; Korrer, M.; Wang, Y.; et al. Immunostimulatory Cancer-Associated Fibroblast Subpopulations Can Predict Immunotherapy Response in Head and Neck Cancer. *Clin. Cancer Res.* **2022**, *28*, 2094–2109 [22]	scRNAseq	Primary tumor	8	22,906
GSE140042	2021	—	scRNAseq	Primary tumor Lymph node metastasis	9	—
GSE200996	2022	Luoma, A.M.; Suo, S.; Wang, Y.; Gunasti, L.; et al. Tissue-resident memory and circulating T cells are early responders to pre-surgical cancer immunotherapy. *Cell* **2022**, *185*, 2918–2935.e29 [23]	scRNAseq + scTCR	Peripheral and intra-tumoral CD45+ populations	204	74,557
GSE153559	2020	Wieland, A.; Patel, M.R.; Cardenas, M.A.; Eberhardt, C.S.; et al. Defining HPV−specific B cell responses in patients with head and neck cancer. *Nature* **2021**, *597*, 274–278 [24]	scRNAseq	B cells Primary tumor Lymph node metastasis Peripheral tumor	7	8271
GSE180268	2021	Eberhardt, C.S.; Kissick, H.T.; Patel, M.R.; Cardenas, M.A.; et al. Functional HPV−specific PD-1(+) stem-like CD8 T cells in head and neck cancer. *Nature* **2021**, *597*, 279–284 [25]	scRNAseq	TILs from primary tumor Lymph node metastasis	39	—
GSE162025	2020	Liu, Y.; He, S.; Wang, X.L.; Peng, W.; et al. Tumour heterogeneity and intercellular networks of nasopharyngeal carcinoma at single cell resolution. *Nat. Commun.* **2021**, *12*, 741 [26]	scRNAseq + scTCR	Primary tumor Peripheral blood leucocytes	40	176,447
GSE150321	2020	Song, L.; Zhang, S.; Yu, S.; Ma, F.; et al. Cellular heterogeneity landscape in laryngeal squamous cell carcinoma. *Int. J. Cancer* **2020**, *147*, 2879–2890 [19]	scRNAseq	Primary tumor	2	12,985
GSE213047	2022	Lin, M.; Sade-Feldman, M.; Wirth, L.; Lawrence, M.S.; et al. Single-cell transcriptomic profiling for inferring tumor origin and mechanisms of therapeutic resistance. *NPJ Precis. Oncol.* **2022**, *6*, 71 [27]	scRNAseq	Primary tumor Normal tissue Lymph node metastasis	3	11,470
GSE172577	2021	Peng, Y.; Xiao, L.; Rong, H.; Ou, Z.; et al. Single-cell profiling of tumor-infiltrating TCF1/TCF7(+) T cells reveals a T lymphocyte subset associated with tertiary lymphoid structures/organs and a superior prognosis in oral cancer. *Oral Oncol.* **2021**, *119*, 105348 [28]	scRNAseq	Primary tumor	6	—

## Data Availability

No new data were created in this study. All the data reported in this review were found in original articles cited in the text.

## Abbreviations

HNSCC	Head and Neck Squamous Cell Carcinoma
scRNAseq	Single-Cell RNA Sequencing
HNCs	Head and Neck Cancers
TME	Tumor Microenvironment
OS	Overall Survival
TCGA	The Cancer Genome Atlas
CPTAC	Clinical Proteomic Tumor Analysis Consortium
OPSCC	Oropharyngeal Squamous Cell Carcinoma
TCR	T Cell Receptor
TILs	Tumor-Infiltrating Lymphocytes
HPV	Human Papilloma Virus
TAMs	Tumor-Associated Macrophages
SC	Single Cell
GEO	Gene Expression Omnibus
CAFs	Cancer-Associated Fibroblasts
DE	Differential Expression
CCC	Cell–Cell Communication
DTR	Differentiation Trajectory Reconstruction
CNV	Copy Number Variation
GO	Gene Ontology
CSCs	Cancer Stem Cells
EMT	Epithelial–Mesenchymal Transition
TNF	Tumor Necrosis Factor
pDCs	Plasmacytoid Dendritic Cells
PNI	Perineural Invasion
MDSCs	Myeloid-Derived Suppressor Cells
PD-1	Programmed Cell Death Protein 1
PD-L1	Programmed Cell Death Ligand 1
MARGs	Methylation/Autophagy-Related Genes
FGFR	Fibroblast Growth Factor Receptor
pRS	Prognostic Risk Scoring System
MCs	Mast Cells
PFS	Progression-Free Survival
TFH	CD4+ T Follicular Helper
DRGs	Cell Differentiation-Related Genes
tsCAFs	T Cell-Stimulating Cancer-Associated Fibroblasts
ICT	Induction Chemotherapy
ICB	Immune Checkpoint Blockade
GC	Germinal Center
MOC	Murine Oral Carcinoma
KO	Knockout
